# Assessing the implementation of national sodium reduction policies in Nigeria: an interim qualitative evaluation of stakeholder perspectives

**DOI:** 10.3389/fnut.2026.1704402

**Published:** 2026-03-26

**Authors:** Chisom Obiezu-Umeh, Vanessa Alfa, Clementina E. Okoro, Ikechukwu A. Orji, Ucheoma Nwaozuru, Linda V. Van Horn, Erica L. Jamro, Nanna Ripiye, Gabriel L. Shedul, Emmanuel I. Okpetu, Adedayo Ojo, Henry Ekechi, Morenike Alex-Okoh, Anyaike Chukwuma, Aloysius N. Maduforo, Felix Adurosakin, Deborah Odoh, Malau Mangai Toma, Alayo Sopekan, Uduak Uwakmfon, Doris John, Guhan Iyer, Bruce Neal, Alexandra Jones, Kathy Trieu, Matti Marklund, Maoyi Tian, Mark D. Huffman, Dike B. Ojji, Lisa R. Hirschhorn

**Affiliations:** 1 Department of Medical Social Sciences, Northwestern University Feinberg School of Medicine, Chicago, IL, United States; 2 Center for Dissemination and Implementation Science, Northwestern, University Feinberg School of Medicine, Chicago, IL, United States; 3 Cardiovascular Research Unit, University of Abuja Teaching Hospital, Abuja, Nigeria; 4 Federal Capital Territory Primary Health Care Board, Abuja, Nigeria; 5 Department of Implementation Science, Wake Forest University School of Medicine, Winston-Salem, NC, United States; 6 Department of Preventive Medicine, Northwestern University Feinberg School of Medicine, Chicago, IL, United States; 7 Cardiovascular Division and Global Health Center, Washington University in St. Louis, St. Louis, MO, United States; 8 Department of Public Health, Federal Ministry of Health, Abuja, Nigeria; 9 Department of Nutrition and Dietetics, University of Nigeria, Nsukka, Nigeria; 10 Department of Community Health Sciences, University of Calgary, AB, Canada; 11 Noncommunicable Disease Division, Federal Ministry of Health, Abuja, Nigeria; 12 The George Institute for Global Health, University of New South Wales, Sydney, NSW, Australia; 13 Welch Center for Prevention, Johns Hopkins Bloomberg School of Public Health, Baltimore, MD, United States; 14 School of Public Health, Harbin Medical University, Harbin, China; 15 Department of General Practice, The Second Affiliated Hospital of Harbin Medical University, Harbin, China; 16 School of Public Health, Washington University in St. Louis, St. Louis, MO, United States; 17 Department of Internal Medicine, Faculty of Medicine, University of Abuja, Abuja, Nigeria; 18 Robert J Havey Institute for Global Health, Feinberg School of Medicine, Chicago, IL, United States

**Keywords:** dietary policy, Nigeria, policy implementation science, process evaluation, sodium

## Abstract

**Introduction:**

Population-wide dietary sodium reduction is a global priority and a highly cost-effective strategy to lower blood pressure and reduce cardiovascular disease risk. In response, the Nigerian government launched the National Multi-sectoral Action Plan (NMSAP) in 2019, recommending evidence-based policy actions including limiting sodium in processed foods, restricting food advertising especially for children, promoting public awareness, integrating nutrition education in schools, and front-of-package labeling. We conducted a qualitative study to examine progress, barriers, and facilitators in implementing these policy actions 3 years post-launch.

**Methods:**

Between March and September 2024, we conducted 47 key informant interviews and 5 focus group discussions with 22 participants from sectors relevant to sodium reduction, including government, education, food manufacturing and retail, civil society, non-governmental organizations and consumers. Guided by the Integrated Theoretical Framework, we identified determinants influencing policy implementation. Three coders independently coded the data, which were thematically analyzed.

**Results:**

Policy characteristics that facilitated implementation included emerging nutrient profiling and dietary surveillance systems and ongoing nutrition education; however, limited baseline data, the absence of nationally endorsed front-of-pack labeling frameworks, and unreliable labeling impeded progress. Within inter-organizational relationships, challenges in coordination, governance, and cross-sector communication emerged as overarching barriers to implementation. Implementing agency capacity was constrained by inconsistent enforcement, limited funding, workforce turnover, low awareness of national sodium policies, and resistance from food manufacturers. Regarding attributes and responses of those affected by the policy, strong support for educating children as agents of household change facilitated school-based nutrition education, although entrenched cultural norms around salt use, financial constraints, and limited public knowledge of the health risks of excessive salt consumption hindered broader behavior change. Finally, within the policy context and external environment, the existing use of local herbs and traditional potassium-rich flavorings was identified as a promising opportunity to promote lower-sodium diets.

**Conclusion:**

These findings offer critical insights for enhancing implementation, sustainability, and scale-up of evidence-informed policy actions in Nigeria and in other low- and middle-income countries.

**Clinical trial registration:**

Trial Registration: NCT04765865.

## 1 Introduction

Unhealthy diet and high-sodium intake are major, modifiable risk factors for hypertension, cardiovascular disease (CVD) and related complications globally (1–3). In Nigeria, as in many other countries, adults consume nearly twice the World Health Organization’s (WHO) recommended level of dietary sodium of 2 g/day (<85 mmol) (4, 5). Reducing excess dietary sodium intake in the general population is widely recognized as one of the most cost-effective strategies to address the global burden of CVD (2, 6). Current evidence suggests that a daily reduction of 2.5 g in sodium intake can lower systolic blood pressure by up to 6.8 mm Hg, potentially reducing the incidence of CVD and all-cause mortality by up to 12 and 7.5%, respectively (1, 7, 8).

Implementing comprehensive, multicomponent, and mandatory policies is an effective approach to reducing population-level sodium intake, such as policies to reshape the structural environments in which individuals make dietary decisions. Such approaches have demonstrated greater impact than interventions targeting individual behavior change alone (9–11). In response to the increasing burden of non-communicable diseases, the Nigerian government launched the National Multi-Sectoral Action Plan (NMSAP) for the Prevention and Control of Non-Communicable Diseases in 2019, which incorporates evidence-based sodium reduction strategies aligned with the WHO SHAKE package (4, 12). The four core priority actions recommended include (1) limiting the amount of salt in prepared and packaged foods; (2) restricting food advertising, particularly to children, to promote healthier diets; (3) conducting public health campaigns to raise awareness about healthy eating and counteract marketing of unhealthy foods and beverages; and (4) integrating nutrition education in schools to ensure children understand how to make healthier food choices (12). Two additional strategies—front-of-package warning labeling (FOPL) and encouragement of low-sodium, potassium-enriched salt substitutes—are also recommended to strengthen the effectiveness of these core actions and support the shift toward healthier, lower-sodium diets (12, 13).

However, in low- and middle-income countries (LMICs) like Nigeria, progress toward implementing these policy actions has been modest, and their potential public health impact remains largely unrealized (13–16). Although recent national initiatives, such as the development of Nigeria’s National Guidelines for Sodium Reduction (17), represent important steps forward, effective and sustained implementation requires active stakeholder engagement, local ownership, alignment of government and donor priorities, widespread adoption, and robust systems for ongoing monitoring and evaluation (18). Policy implementation science offers a valuable lens through which researchers, practitioners, and policymakers can examine how such policies are translated into practice, determine whether they achieve intended outcomes, and identify factors that influence implementation success (19, 20). While several studies have examined the health outcomes associated with sodium reduction policies, particularly in relation to hypertension and incident CVD, relatively few have focused on evaluating the processes and outcomes of policy implementation (9, 16).

To address this gap, the Nigeria Sodium Study (NaSS) was initiated to evaluate the implementation and scale-up of the NMSAP policy actions aimed at reduction of dietary salt intake in Nigeria. Guided by the Exploration, Preparation, Implementation, and Sustainment (EPIS) framework and employing a mixed-methods design, NaSS includes three waves of stakeholder interviews, food retail surveys, and population surveys conducted across three regions: the Federal Capital Territory (North-Central), Kano State (North-West), and Ogun State (South-West). The first wave of interviews (January–February 2021), corresponding to the exploration and preparation phases of EPIS, assessed stakeholders’ knowledge, attitudes, and perceived barriers to implementing the core NMSAP policy actions (21). Barriers emerged at the individual (limited awareness, distrust in government, cultural food practices), organizational (poor label design, fear of customer loss), and policy (poor advertising regulation, bureaucracy within organizations and affordability of healthier alternatives) levels (21). However, stakeholders’ perspectives on the uptake of low-sodium, potassium-enriched salt substitutes were not explored during this formative phase.

Building on these early findings, the current study presents results from the second wave of data collection. Using Bullock et al.’s Integrated Theoretical Framework for understanding policy implementation (22), the study evaluates implementation progress and examines multilevel contextual factors influencing the implementation of NMSAP’s sodium reduction strategies including front-of-package labeling and low-sodium, potassium-enriched salt substitutes. These insights aim to not only inform strategies for enhancing the uptake, sustainability, and scale-up of NMSAP policy actions, but also to contribute to a growing body of knowledge on implementing and sustaining nutrition-related policy interventions in Nigeria and other LMICs.

## 2 Methods

### 2.1 Study design

As part of the implementation phase of EPIS, this qualitative evaluation was conducted between March and September 2024 during the second wave of the Nigeria Sodium Study (NaSS; ClinicalTrials.gov registration: NCT04765865). Data were collected through in-depth interviews with key informants and focus group discussions with consumers. All procedures were approved by institutional review boards at University of Abuja, Northwestern University, Washington University, and the University of New South Wales. All participants provided written informed consent for participation. The Consolidated Criteria for Reporting Qualitative Research (COREQ) (23) guided data analysis and reporting.

### 2.2 Conceptual framework

The Integrated Theoretical Framework by Bullock and colleagues was used as the underlying theory for analysis of findings (22). This framework was selected to address prior critiques of policy implementation research in low- and middle-income countries, particularly the need for better conceptualization of the multi-level and multi-sectoral complexities that characterize policy contexts in these settings (24, 25). The framework identifies eight key determinants influencing policy implementation: (a) policy characteristics, (b) policy formulation process, (c) vertical administration and thickness of hierarchy, (d) inter-organizational networks and relationships, (e) implementing agency responses, (f) attributes and responses of those affected by the policy, (g) timing and sequencing, and (h) external environment or broader policy context.

### 2.3 Study setting and participants

Participants held senior-level positions or were representatives within five categories of organizations involved in the implementation, research, policy development, regulation, or promotion of sodium reduction efforts: (1) government agencies at the federal, state, or local levels; (2) non-governmental organizations, including international, public health professional bodies and civil societies; (3) education sectors; (4) food manufacturers and retailers; and (5) consumers. We employed maximum variation sampling to ensure diversity in organizational representation and stakeholder roles in NMSAP implementation and policymaking across three geographic regions: Federal Capital Territory, Kano State, and Ogun State. These locations were selected due to their active involvement in the ongoing pilot implementation of selected NMSAP priority actions by the Nigerian Federal Ministry of Health and WHO.

Stakeholders were identified with support from the authors (DBO, AO, and CEO), who have extensive experience in Nigeria’s health policy landscape and stakeholder engagement (13, 15). Where possible, we re-engaged participants from the first wave of interviews; however, some were replaced due to changes in position within the organization. Invitations were sent via email and formal letters. Additional participants were identified through snowball sampling during interviews. Consumers were recruited from primary health centers and community spaces within the catchment areas via flyers and referrals. Eligibility criteria for consumers included: (i) being aged 18 years or older, and (ii) providing informed consent for interview and audio recording. The final sample size of the key informants and consumers was determined by data saturation until no new themes emerged.

### 2.4 Data collection

Semi-structured, open-ended interview and focus group guides were developed and pilot tested for each participant group. The focus group guides were initially developed in English which is Nigeria’s national language. The in-depth interviews were conducted with key informants and policymakers to elicit detailed, individual insights, leveraging their expertise and current roles. In contrast, focus groups were conducted with consumers to capture collective lived experiences, allowing participants to compare perspectives and generate community-level insights that may not emerge in individual interviews.

The key informant interview guide explored participants’ perceptions on the policy contents, successes, challenges, facilitators, and recommendations related to the implementation of the four NMSAP priority actions as well as front-of-package labeling and use of salt substitutes. While the guide included a core set of questions applicable to all informants, additional questions were tailored to each participant’s organization type and specific role. Focus group discussions with consumers explored their understanding, attitudes, behaviors, and lived experiences related to dietary sodium consumption and exposure to related policy actions.

In-person interviews were conducted at the participant’s office or another convenient location at their workplace, while focus groups were held in a neutral, centrally located meeting space to facilitate travel and attendance. All interviews and focus groups sessions were conducted by trained study team members with extensive qualitative research experience, including moderating group discussions. Sessions were conducted in English or Pidgin English, audio-recorded, and lasted approximately 45 to 60 min. Before each session, participants were briefed on the study objectives and confidentiality procedures. Audio recordings were transcribed verbatim, translated into English where necessary, and reviewed for accuracy.

### 2.5 Data analysis

Thematic analysis was used to identify and summarize common themes from the interviews. Although the eight determinants from the integrated framework guided the overall organization of the themes, we also employed an inductive, emergent, open-coding approach, allowing themes to arise directly from the data without predetermined categorization. In line with best practices, three researchers (CO, VA, and UN) independently double-coded a subset of five transcripts (10% of the full set of transcripts) to identify preliminary codes and ensure consistency in coding (26). The team met weekly to compare coding and resolve discrepancies, with consultations from a fourth researcher (LRH) to resolve any discrepancies and reach consensus. The remaining transcripts were then evenly divided among coders. All coding processes were performed using Dedoose software (version 9.2.5 Los Angeles, CA: SocioCultural Research Consultants, LLC). After coding was completed, the main coder (CO) generated a coding report, organized findings into matrix displays using Microsoft Excel, and categorized themes under the relevant constructs of the integrated framework for each stakeholder group, indicating whether each represented a barrier (−), facilitator (+), or both (−/+). Matrices for each group were then compared to determine cross-cutting themes and explore differences across groups.

## 3 Results

As shown in Table 1, a total of 69 stakeholders participated in the study, including 47 key informants and 22 participants in five focus group discussions. The median age was 46 years (IQR: 37.5–55.0), and most were male (60.3%). Over half of the participants were officials from local, state, or federal government agencies (50.6%), with most based in the Federal Capital Territory (95.8%).

**Table 1:** Participant characteristics.

Characteristics	No. of participants (*N* = 69)		
	IDI (*n* = 47)	FGD (*n* = 22)	Total
Age, median (IQR), y	52.5 (42.5, 56.8)	30.0 (24.0, 42.0)	46.0 (37.5, 55.0)
Sex, *n* (%)			
Female	17 (38.3)	10 (45.0)	27 (39.7)
Male	29 (61.7)	12 (55.0)	41 (60.3)
Organization type			
Local, state and federal government	30 (63.8)	5 (22.7)	35 (50.7)
Non-governmental organization*	10 (21.3)	0 (0.0)	10 (14.5)
Education sector	3 (6.4)	0 (0.0)	3 (4.3)
Food manufacturing and retail	4 (8.5)	0 (0.0)	4 (5.8)
Consumer	0 (0.0)	17 (77.3)	17 (24.7)
Participants location			
Federal Capital Territory	45 (95.8)	22 (100.0)	67 (97.2)
Kano	1 (2.1)	0 (0.0)	1 (1.4)
Ogun	1 (2.1)	0 (0.0)	1 (1.4)

IDI, in-depth interviews; FGD, focus group discussion; CHO, community health officers; CHEW, community health extension workers; IQR, Interquartile range. *Non-governmental organizations, including international, public health professional bodies and civil societies.

Qualitative findings mapped onto five of the eight policy implementation determinants outlined in the integrated framework: policy characteristics, inter-organizational networks and relationships, implementing agency responses, attributes and responses of those affected by the policy, and external environment or broader policy context. Policy formulation was not explored in this analysis, as it had been addressed during the formative phase of NaSS (21). The domains of vertical administration and hierarchy and timing/sequencing were also excluded due to insufficient data related to these determinants.

### 3.1 Policy characteristics

This theme refers to the design, content and contextual fit of the sodium reduction policies, along with related regulatory tools that shape how effectively these policies are implemented and adopted. Themes and illustrative quotes are presented in Table 2.

**Table 2:** Themes and illustrative quotes on policy characteristics.

Theme	Valence	Illustrative quotes
Building nutrient profiling and surveillance systems	+	“The one that I am fully aware of is the nutrition profiling system that is ongoing. It’s a step in the right direction to guide people on how good or how safe whatever they are consuming is especially for processed foods.” [IDI, Federal government representative] “There is steps survey which will enhance the surveillance for us to understand the level, the pattern of consumption, the level in the different foods.” [IDI, International NGO]
Ongoing nutrition education and public health campaigns	−/+	“Well, at an individual scale, I’ve seen quite a number of social media campaigns. There’s one by one guy I think I’ve seen him talking about the effects of salt. There’s also a campaign that was led by the First Lady of Anambra State on salt. So, I mean, I think individuals and small organizations have been going on with campaigns on sodium reduction, but I would not really say it’s quite massive.” [IDI, International NGO] “Recently we have been able to introduce NCD clinic days at the PHC levels, where there will be public health education on NCD and risk factors which include healthy diet, that is what I am aware of and that is presently currently going on in some of our pilot sites like in Ogun and Kano.” [IDI, Federal government representative]
Existing school-based nutrition programs	−/+	“In schools, you have nutrition, foods, and nutrition, as a subject and of course, it talks about the food quality, the food content, and so on and so forth. So, that has been ongoing for a long time.” [IDI, State government representative] “I know that recently fruits day, water day, they have been trying to promote that in schools, but it has not really been effective. It has only been adopted in a few schools, which are mainly private schools. There have been efforts here and there, but it has not been nationally accepted.” [IDI, Federal government representative]
Limited data and evidence to support regulation	−	“I think unavailability of data might also have been an issue. And a lot cannot really be done if the data is not available. If you talk about sodium target setting, you really need data to come up with national targets. If you even start talking about nutrient profile modeling, you also need available data as well. So, perhaps, if there were existing data in place prior to these documents, maybe things would have moved…There’s no evidence to show that whatever is seen in the food is causing the populace to consume excess salt than recommended by the WHO. So, if this does not happen, I really do not expect the manufacturers to reduce the salt, because, I mean, there’s no complaints anyway” [IDI, International NGO] “I think there’s need to give us data that say at least this is the maximum you can use for a bag. I think what will give success to this thing is if we can know the limit, then we use that one to campaign and let people know that at least, for example 700 is the highest that you can use, so people will find a way to adjust.” [IDI, food manufacturer/retailer]
Guiding framework for front-of-pack labeling practices	−	“NAFDAC cannot go into front of pack, back of Pack labeling, only when we have decided which policy we are following. Is it the WHO policy or the nutritional policy of the UK? It must be dependent on one nutrition theory. And once it is established, then it will be easy to follow that model, nutritional model, to do the front of back or back of pack. We are not going to reinvent the nutritional model. Because if you force somebody to do front of pack or back of pack, if NAFDAC wake up today and say you must do front of pack, one industry can just challenge you based on which policy of the federal government. And at the end to determine that case you cannot implement so we need a nutritional model defined by appropriate ministry saying that this is the Nigerian adopted nutritional level then every other sector like NAFDAC must be complied by.” [IDI, Federal government representative]
Reliability and design of nutritional labels	−	“Usually every product, they have not been doing front of pack labeling. If you check the back of pack labeling, you will see a breakdown of the composition of that item and that breakdown; understanding is a problem for most people, especially uneducated people. And it does not give a correct guide; some might see that it contains a certain milligram of salt or anything; that does not tell you whether it’s safe or not.” [IDI, Federal government representative] The content place is so tiny. They are usually difficult to understand. They are always hiding it” [FGD, Consumer]
Limited nutritional labeling coverage on locally manufactured products	−	“Apart from foreign goods that come in. I do not think we put such information [i.e., FOPL] on our own manufactured products.” [IDI, Federal government representative] “How many of us are actually buying foods that are labeled, the majority of the population do not buy labeled food when you buy from the open market? So, we are not there yet.” [IDI, Federal government representative]
Potential taste differences between salt substitutes and regular salt	−	“If the taste does not change the quality or the taste of their food, I think it will be acceptable.” [IDI, State government representative] “Most people believe that if you prepare the same soup, one with sodium salt, and another with potassium salt, that the taste will differ. Some people do not like the taste of potassium salt; they prefer not to even have it in their meal.” [IDI, Federal government representative]
Higher price of salt substitute compared to regular salt	−	“If salt is costing fifty Naira and you bring this because it’s a salt substitute and you are telling me to buy it for five hundred Naira, people will tell you, I’d rather stick to my salt.” [IDI, Local government representative] “Like some of these substitutes, some people might tell you that,‘Oh, it’s a bit expensive, when you have it you might not really feel it, you need to add a good quantity of it in your meal before you can feel that effect or make your food tasty.’ So, people will tell you they need money to buy this substitute because some of them are very expensive, and you need more quantity in your meal.” [IDI, Federal government representative]

+ indicates facilitator or positive progress, (−) represents challenge or limited implementation; NAFDAC, National Agency for Food and Drug Administration and Control; IDI, in-depth interviews; FGD, focus group discussion; NGO, Nongovernmental Organizations; NCD, Non-communicable disease.

#### 3.1.1 Building nutrient profiling and surveillance systems

Several federal-level policymakers and international NGO representatives described ongoing efforts to establish a national nutrient profiling system and strengthen dietary surveillance as promising steps toward reducing excess dietary sodium intake. A nutrient profiling system was viewed as a key tool for guiding consumers toward healthier food choices and promoting standardized labeling practices among food manufacturers. Respondents also emphasized the value of surveillance efforts, such as the ongoing STEPS (STEPwise approach to noncommunicable disease risk factor surveillance) survey, in generating data on dietary patterns and sodium levels in the food supply chain, which were perceived to be key for facilitating implementation and monitoring of sodium reduction policies.

#### 3.1.2 Ongoing nutrition education and public health campaigns

Participants described that public awareness around sodium reduction was being promoted through various health education efforts delivered across multiple platforms, including community outreach, clinical counseling, and school-based health talks. Specific activities mentioned included health talks in communities, educational sessions during NCD clinic days at primary health centers, and clinician-led dietary counseling. Social media and individual-led initiatives, including those led by health influencers/promoters, were also cited as important avenues for disseminating sodium-related information. While these efforts reflect progress in implementing public health campaigns, participants noted that such campaigns were often limited in scale and primarily targeted individuals already diagnosed with hypertension, rather than broader, preventive outreach to the general population.

#### 3.1.3 Existing school-based nutrition programs

Nutrition education was reported to be part of the existing school curriculum, with courses like “Food and Nutrition” educating school children about food quality and content, an opportunity that respondents felt could be leveraged to promote sodium reduction in school-based settings. Additionally, programs like the National Homegrown School Feeding Program and “Fruits Day” were cited as ongoing efforts to encourage healthier food choices among school children. However, respondents also noted that some of these initiatives were often limited to select, primarily private schools, and had yet to achieve widespread or consistent national implementation, largely due to resource constraints and limited government support.

#### 3.1.4 Limited data and evidence to support regulation

While there has been progress in developing nutrient profiling and surveillance systems, several respondents noted that limited baseline data on sodium intake and levels in packaged foods has hindered the implementation and adoption of the policy actions since the launch of NMSAP. Such data were considered essential for setting reduction targets, establishing a national nutrient profiling system, and supporting regulatory enforcement. They also cited limited evidence on the main sources and quantities of sodium in commonly consumed foods. In the absence of such data, alongside little-to-no consumer complaints and regulatory pressure, food manufacturers were perceived as unlikely to voluntarily reformulate products or reduce sodium content.

#### 3.1.5 Guiding framework for front-of-pack labeling practices

Adoption and implementation of FOPL has been limited in part by the absence of nationally endorsed guiding principles and a framework adapted to the Nigerian context. Participants noted that without clearly defined national standards and guidelines, labeling mandates would likely face resistance from food manufacturers, thereby delaying widespread implementation and uptake.

#### 3.1.6 Reliability and design of nutritional labels

Participants raised concerns regarding the reliability of nutritional information on food labels, citing weak quality control measures at the manufacturing level and the potential for inaccurate representation of food content, which in turn undermines consumer trust in labeling and limits its effectiveness in sodium reduction. They also noted that labels were often difficult to read or understand due to small print, technical language, or lack of contextual interpretation (i.e., what levels are considered healthy).

#### 3.1.7 Limited nutritional labeling coverage on locally manufactured products

Among the few participants (mostly those from federal-level agencies and international NGOs) familiar with FOPL, it was more commonly observed on imported packaged products. Respondents noted that locally manufactured foods often lack standardized nutritional information. They further described that a significant portion of the population purchases unpackaged foods from open markets, where labeling practices are not standardized and thus suboptimal, limiting the reach and effectiveness of labeling strategies for sodium reduction.

#### 3.1.8 Potential taste differences between salt substitutes and regular salt

Potential differences in taste between salt substitutes and regular salt were perceived as a barrier to their acceptability. Participants noted that salt substitutes would be more acceptable if they did not alter the taste or quality of food; however, many respondents perceived these substitutes as less palatable. A few respondents also believed that larger quantities of salt substitutes might be needed to achieve a flavor profile similar to that of regular salt.

#### 3.1.9 Higher price of salt substitute compared to regular salt

Several participants raised concerns about the affordability of salt substitutes, which were perceived to be higher in price than regular salt, thereby limiting accessibility. While the long-term health benefits were acknowledged, respondents emphasized that for most consumers, particularly those with limited financial resources, immediate affordability outweighs health considerations, influencing the reach and acceptability of salt substitutes.

### 3.2 Inter-organizational networks and relationships

The theme captures the nature and quality of coordination, collaboration, and communication across government ministries, departments, agencies, and other stakeholders involved in the implementation of sodium reduction policies. Themes and illustrative quotes are presented in Table 3.

**Table 3:** Themes and illustrative quotes on inter-organizational networks and relationships.

Theme	Valence	Illustrative quotes
Coordination, governance, and cross-sector communication	−/+	“The issue of coordination and governance. I mean, in carrying out interventions like this, if there’s really no meeting points where everyone working in the same space comes together to align on priorities, align focus, align on interventions, you will notice duplication of activities, you will notice information loss…there will not be efficiencies in the use of resources. So, what this means is that probably an action that would have taken 2 years ends up taking 5 years because there’s poor coordination and poor governance; Where the party A does not even know what B is doing and where B does not even know what C is doing. Where there’s really no forum for stakeholders to come together and spread brainstorm over roadblocks and be able to overcome it, that could be an issue.” [IDI, International NGO] “We have already put structures in place by setting up technical working groups on reducing salt intake. The technical working group is part of the coordination mechanism for the multi-sectoral action plan.” [IDI, Federal government representative]
Unclear roles and delegation of responsibilities	−	“When this multi-sectorial action plan was out, responsibilities were not well spelled out for the parties.” [IDI, Federal government representative] “Each ministry, agency, department needs to know their roles and responsibility.” [IDI, Federal government representative]

+ indicates facilitator or positive progress, (−) represents challenge or limited implementation; IDI, in-depth interviews; NGO, Nongovernmental Organizations.

#### 3.2.1 Coordination, governance, and cross-sector communication

Several participants identified coordination, governance, and cross-sector communication as areas requiring further strengthening to support effective implementation. Respondents noted that without a centralized platform or mechanism to align priorities, share information, and coordinate efforts, agencies and organizations often work in silos, leading to duplication of efforts, inefficiencies, and missed opportunities for synergy. Participants from various sectors acknowledged limited visibility into the activities of others, making it difficult to track progress or identify collective impact. Despite these challenges, a few respondents highlighted recent structural developments, such as the establishment of technical working groups focused on sodium reduction, as important steps toward improving multisectoral coordination and enhancing implementation efforts.

#### 3.2.2 Unclear roles and delegation of responsibilities

Participants highlighted some uncertainty around roles and responsibilities across ministries, departments, and agencies. They also noted that the absence of detailed, sector-specific action plans and clear timelines posed challenges to the effective rollout of sodium reduction policies, limiting implementation progress.

### 3.3 Implementing agency responses and attributes

The theme captures the capacity, commitment, and characteristics of government entities and implementing agencies that influence their ability to implement the sodium reduction policies. Themes and illustrative quotes are presented in Table 4.

**Table 4:** Themes and illustrative quotes on implementing agency responses and attributes.

Theme	Valence	Illustrative quotes
Existing regulations of food advertising and public messaging	−/+	“We control advertisements of repackaged goods. And there is already a whole division handing advertisement permits. So, the way to achieve that is that the NAFDAC division handles this permit. They do not just grant permits for advertising either audio or visual. What they do is that it must undergo certain process and once your submission, your video or your script is vetted and things that are obnoxious or offensive to children and nutrition. They will not be permitted even if they are granted, such portions should have been removed or amended. And it is working effectively. It is still one of the basic procedures before advert of any food product, and it is more serious when it concerns children targeted foods.” [IDI, Federal government representative] “I do not think there is progress, because for instance, we still see them [unhealthy food products] on TV advertising for kids, you see the drinks, you see all these foods that they advertise. And because, of course the media or the Television, they want to make money, it’s business.” [IDI, Federal government representative]
Inconsistent regulatory enforcement	−	“Weak regulatory systems affect almost every area in this country. There might be policies everywhere but implementing it, follow-up, regulating it is another thing.” [IDI, Federal government representative] “In Nigeria, we have a lot of policies. But implementation becomes a problem. We package a lot of fantastic documents, and we keep them under the cupboard, without implementing it. You’ll need to follow them by enforcing them.” [IDI, State government representative]
Leadership support and commitment	−	“A big factor could be leadership. If there’s no political will from the government to implement it, it will not go far.if the government does not have resources and there is also no appetite among, probably, partners or other intergovernmental organizations to support this process, it might also stall.” [IDI, International NGO] “Some agencies think that NCD should not be their priority, and so not wanting to put enough budgetary line to the implementation of the plan.” [IDI, Federal government representative]
Insufficient funding and resources constraints	−	“Everything is about funding. Even on my schedule, we have as part of our activities to carry our sensitization in schools, but you find out that, somehow you start it, you end up funding it from your pocket, and at a time you get tired of it, and you back out. Only very few, and those few struggling to get funds and even when they do some pilot projects, the sustainability of it that government is actually supposed to take over still does not happen.”[IDI, State government representative] “And then, apart from that, when you come to the government side, most at times, government funding programs or activities is always difficult. Mostly, it’s donor driven. And then, when the donors now come to take up activities, or bring up a program, once they conclude and then they while away, the program dies off.” [IDI, Local government representative]
Workforce turnover and shortages	−	“Part of the barriers also is staff attrition. By the time you train focal persons in these ministries in the area of multi-sectoral action plans for NCD control and control of NCD risk factors; and then later the staff might be transferred, you would have to retrain again, So, before you train again, you know, some staff may retire. This has also been frustrating efforts.” [IDI, Federal government representative] “Part of the barrier is the lack of health educators in our schools. We do not have health educators in schools; I think what we have in most schools is maybe the school nurse, the clinic. But are they playing this role of education on healthy diet.” [IDI, Federal government representative] “The number of teachers is inadequate. We do not have many food and nutrition teachers, qualified teachers.” [IDI, Federal government representative]
Monitoring and accountability mechanisms	−	“We should have an agency that monitors to make sure that what we are putting. If you say sodium is 5 grams or 10 grams, that’s true. Because we have a lot of manufacturers that give us the wrong information just for us to buy. Because there is no monitoring agent to say, ‘Yes, this thing is correct.’ So, if we have agencies that are monitoring to make sure that the percentage they are telling us is the correct percentage in that product, it will help us.” [IDI, Federal government representative]
Low organizational awareness of national sodium policies	−	“We can say the intervention has not been optimal because there’ve not been a lot of awareness. I do not think everybody is aware of the NMSAP document yes, so we need to have a lot of awareness especially about the existence of this document.” [IDI, Federal government representative] “Lack of awareness, most of the teachers do not even know about this even the health teachers do not even know about this.” [IDI, Local government representative]
Resistance by food manufacturers and retailers	−	“Well, those companies, what they after is they are after their money. When they advise something that is not going to bring customers to them, to sell much, they will not do it.” [FGD, Consumer] “Pushback by the industry is always there because industries are mostly for making profits. So, anything that will make them reduce their profits, there’s always resistance. And there’s also cost implication if they have to reformulate rather than put warning label, then, of course, they will want to run away from it. So, these are the pushbacks that we end up with.” [IDI, International NGO]

+ indicates facilitator or positive progress, (−) represents challenge or limited implementation; NAFDAC, National Agency for Food and Drug Administration and Control; IDI, in-depth interviews; FGD, focus group discussion; NGO, Nongovernmental Organizations; NMSAP, National Multi-Sectoral Action Plan.

#### 3.3.1 Existing regulations of food advertising and public messaging

While some respondents expressed concerns over the inadequate regulation of food advertising, particularly the promotion of unhealthy foods to children via television and other media, others acknowledged that regulatory structures are in place to support implementation of policies restricting food advertising. Federal-level government representatives described systems to review advertisements and restrict misleading content, especially for products targeting children. However, enforcement was commonly characterized as inconsistent, with commercial interests often outweighing public health priorities. Several respondents drew parallels to past successes in restricting tobacco advertising and called for similar measures (i.e., loss-framed messaging to communicate health risks) to regulate food marketing directed at children. They also emphasized the need for collaboration between the health sector and other influential sectors, such as media and communications, to strengthen the implementation of restrictions on unhealthy food advertising to children.

#### 3.3.2 Inconsistent regulatory enforcement

Inconsistent enforcement of policies across different levels of government was cited as a barrier to implementing sodium reduction policies. Participants also described a generally under-funded and thus fragile regulatory system across sectors, where implementation of existing policies are undermined by inadequate follow-up and enforcement, limiting their effectiveness and slowing progress.

#### 3.3.3 Leadership support and commitment

Participants consistently emphasized the importance of leadership support for the successful implementation of sodium reduction policies. However, several described limited leadership and political commitment as a major barrier to progress to date. This lack of commitment was reflected in the low prioritization of sodium reduction and NCDs, inadequate budget allocations at the federal and state levels, and minimal investment in public education campaigns on excess salt intake—all of which were perceived to hinder implementation efforts.

#### 3.3.4 Insufficient funding and resource constraints

Funding and resource constraints emerged as major barriers to the implementation and sustainability of sodium reduction policies and related nutrition programs. Respondents consistently described funding as inadequate, with many initiatives largely driven by donor support. While external partners often played a critical role in initiating efforts, participants noted that monitoring and enforcement activities frequently stalled due to these funding limitations.

#### 3.3.5 Workforce turnover and shortages

Staff attrition within government agencies was identified as a key challenge to workforce stability. Frequent transfers and retirements of personnel trained on the Multi-Sectoral Action Plans for NCD Prevention and Control disrupted continuity and hindered sustained implementation. In school settings, respondents also highlighted a shortage of trained health educators as a barrier to the adoption of nutrition education programs. Many schools relied solely on school nurses or clinics, which often did not actively provide education on healthy diets.

#### 3.3.6 Monitoring and accountability mechanisms

Most participants described a lack of formalized and routine systems for monitoring the accuracy of food labels, including nutrient declarations and claims on packaged foods. They emphasized the need for stronger oversight by federal-level agencies to ensure accurate labeling, including systematic market surveillance, laboratory testing, verification of manufacturer claims, and enforcement of accountability mechanisms such as sanctions for non-compliance.

#### 3.3.7 Low organizational awareness of national sodium policies

Low awareness of the NMSAP sodium-related policies was identified as a major barrier to reach and implementation of these policies. Many respondents noted that even key actors within federal, state and local government agencies were unaware of these policies and related efforts, highlighting significant gaps in dissemination and the need for more targeted engagement.

#### 3.3.8 Resistance by food manufacturers and retailers

Resistance from food manufacturers and retailers emerged as a barrier to policy adoption and implementation. Respondents noted that manufacturers often exhibit risk aversion, particularly when proposed changes—such as product reformulation or the introduction of front-of-package labels, including warning labels—do not offer clear financial benefits. Without mandatory sodium limits, participants described that there is little incentive for manufacturers to alter products that are already performing well in the market. Cost implications of reformulation and fears of reduced consumer appeal were also frequently cited as reasons for this resistance.

### 3.4 Attributes and responses from those affected by the policy

This theme encompasses the knowledge, beliefs, attitudes, and behaviors of individuals and communities affected by sodium reduction policies. Themes and illustrative quotes are presented in Table 5.

**Table 5:** Themes and illustrative quotes on attributes and responses of those affected by the policy.

Theme	Valence	Illustrative quotes
Strong belief in educating children to influence family health behaviors	+	“Children listen to their teachers than their parents. So, when you meet their teacher, and explain to them, right at that age, they will listen more than you, the father and mother. And, at times, if they hear it often, even if they are playing among other children, they’ll bring it. If children are playing, whatever they learn in school and understand, do you know they discuss, if they are playing outside, even in the sand, they are discussing.” [FGD, Consumer] “If these children are taught that when you want to eat something, just look at it, look at the sodium content of it, if it is between this range, it’s good for you. If it is between this range or above this range do not take it. I’m sure the children themselves will even be telling their parents.” [IDI, food manufacturer/retailer] “Catching them young, making them understand, practicing it [healthy diet] from now, and pushing them to do it the right way, will also help. And as they grow, they will also know that in their own family, in their generation, they will carry it on that way.” [IDI, NGO]
Cultural beliefs, norms, and attitudes around salt use	−/+	“I’m used to this thing, why should I change? And as long as they do not see the effect directly on them, most people will not want to change. Okay, I’ve been doing this for a long time and there’s nothing wrong with me, so why should I?” [FGD, Consumer] Like we Africans, we like spicy food. We like spicy food, like I do not know about this Maggi star. Naturally, Maggi star has salt in it. So, after such an intake of Maggi why now add too much salt again in it? So, it’s because we Nigerians like our food to be too spicy, we want to have that taste.” [IDI, Education sector] “I think everybody is becoming conscious of their cooking… And there’s no person either adult or young that wants to die or wants to be paralyzed…Before, when we buy coke [drink], everybody just carries and be drinking anyhow. But now, now because of the level of sugar that is inside it, my wife has now developed drink using *green kunu* (gruel).” [IDI, food manufacturer/retailer]
Financial constraints	−	“You know in Nigeria, people are trying to survive; whatever you see, you eat.” [IDI, health professional] “One of the barriers is poverty. Somebody will buy what he knows he can buy to survive himself. And I use the word poverty in the sense that if I know this thing I’m going to buy is good for me. But unfortunately, because I do not have the means of buying it, I will go for the one that I can afford.” [IDI, Federal government representative]
Limited public knowledge and awareness	−	“There has been no awareness, it has not been publicized; we have not seen it publicly. We only get this awareness when we visit our doctors and if we have any health issues that are related to the intake of salt, that’s when they will say ahh you have been taking a lot of salt; slow it down. But publicly government is not telling us that this is what we need to do to cut down on eating our salt.” [IDI, Education sector] “People do not really know the amount of salt to consume. People do not know the effects of excess salt intake.” [IDI, Federal government representative]
Limited awareness and misperceptions about salt substitutes	−	“I’m not aware, I’m just hearing for the first time. I know bananas have potassium but salt being produced with potassium that we can use. I’ve never.” [IDI, Federal government representative] “Potassium is even more dangerous than sodium from my own point of view. So, if that is the same potassium, then it’s like jumping from frypan to fire. So, it’s not of any benefit. “[IDI, Federal government representative]

+ indicates facilitator or positive progress, (−) represents challenge or limited implementation; IDI, in-depth interviews; FGD, focus group discussion; NGO, Nongovernmental Organizations.

#### 3.4.1 Strong belief in educating children to influence family health behaviors

Early education on healthy dietary practices was perceived as a promising long-term strategy for sodium reduction. Schools were identified as key entry points, with respondents emphasizing that children not only internalize health messages more effectively but can also influence household behaviors. Teaching children about salt intake was viewed as a pathway to intergenerational change, as early exposure could shape future norms and practices. Participants also noted that children tend to trust and share lessons from teachers, making them potential agents of change within families.

#### 3.4.2 Cultural beliefs, norms, and attitudes around salt use

Sociocultural norms and long-standing dietary preferences emerged as key barriers to reducing sodium intake. Respondents described salt use as deeply ingrained in everyday cooking and eating practices, with many associating high salt contents—and the use of seasoning products such as bouillon cubes—with flavor, satisfaction, and cultural norms. Salt was also noted to serve a functional role in food preservation and is often readily available on dining tables, reinforcing habitual overuse. Several participants pointed to consumer skepticism toward dietary change and healthier alternatives, particularly in the absence of immediate or visible health consequences. This perceived lack of urgency, combined with entrenched taste preferences and social norms, was viewed as major challenges to public acceptance of sodium reduction efforts. In contrast, some respondents also described a growing health consciousness, seen in increased use of and interest in healthier alternatives, especially during cooking (e.g., more vegetables, reduced use of red palm oil, less sugar), which may encourage broader acceptance and adoption of low-salt diets and healthier habits.

#### 3.4.3 Financial constraints

Financial constraints were commonly cited as a major barrier to adopting healthier eating behaviors. Respondents explained that limited resources restrict individuals’ ability to choose healthier food options, often constraining consumer agency. As a result, many people consume readily available or more affordable foods, which are typically higher in sodium, even when they are aware of healthier alternatives.

#### 3.4.4 Limited public knowledge and awareness

Limited public awareness and understanding of recommended salt intake levels, how to interpret nutritional labels, and the health risks of excessive salt consumption were frequently identified as barriers to consumer acceptability and motivation for dietary change. Many respondents noted that this knowledge gap reduces the perceived urgency to reduce salt consumption.

#### 3.4.5 Limited awareness and misperceptions about salt substitutes

Many respondents were unfamiliar with low-sodium, potassium-enriched salt substitutes and questioned their safety, with some perceiving potassium as more harmful than sodium—highlighting a key knowledge gap that may hinder their acceptability and adoption.

### 3.5 Policy context and external environment

This theme refers to the broader socio-political, and environmental factors that influence the implementation of sodium reduction policies. Themes and illustrative quotes are presented in Table 6.

**Table 6:** Themes and illustrative quotes on policy context and external environment.

Theme	Valence	Illustrative quotes
Alternative spices and herbal products	+	“Some people use potassium, local potassium, in some of their food. Like in the Northern part of the country, I know that they use potassium to add to their soup like that. And once they do that in the soup, they add potassium, they do not add much salt. Because if you do that, the soup will be too salty, you will not be able to take it.” [IDI, Local government representative] “It is more practiced at the local level. At the rural area, a lot of them are using alternative spices. Even herb and plant products they are using are not as much pronounced in the urban area. They are already making substitutes with different seasonings that are not salt based.” [IDI, Federal government representative]
Government leadership transitions	−/+	“In Nigeria now, every 4 years we have a change of government, let us say there were existing policies that the government is working on you understand? Let us say after 4 years when another government comes in, that is a change in government officials; it will affect because when a new government comes, he wants to bring his own policy, but the existing ones might want to adapt to the new government policy.” [IDI, Education sector]
Limited availability of low-sodium salt substitutes	−	“Availability is a factor that will hinder it. If you are making something available, make it available. There are plenty of products. Let me just use Coca-Cola as an example. They will bring out products. When people start liking the products, then all of a sudden you see that they have disappeared. It’s not on the market again.” [IDI, Local government representative] “Availability will be a key issue because at the moment I’m not sure how many people are importing it. it’s just that availability that I worry about.” [IDI, International NGO]
Proliferation of unhealthy food products	−	“It is now being compounded by so many adulterations of foods and food sources now because they just want to make things appeal to people irrespective of what the actual ingredients are.” [IDI, Federal government representative] “We import most of these processed foods. So, we really cannot detect how much sodium they put in this thing they are bringing. So, when we import them, we consume them as they come.” [IDI, State government representative]

+ indicates facilitator or positive progress, (−) represents challenge or limited implementation; IDI, in-depth interviews; FGD, focus group discussion; NGO, Nongovernmental Organizations; NCD, Non-communicable disease.

#### 3.5.1 Alternative spices and herbal products

Several respondents highlighted the existing use of traditional seasonings and natural alternatives to sodium salt, particularly in rural areas. Local potassium-enriched salts, herbs, and indigenous flavorings were described as common substitutes that enhance taste while reducing reliance on added sodium. These natural options were perceived as flavorful and culturally acceptable, offering a promising avenue for promoting lower-sodium diets.

#### 3.5.2 Government leadership transitions

Changes in government leadership were noted to influence the implementation and sustainability of sodium reduction policies. Respondents noted that with each change in government, existing policies risk being deprioritized or replaced, as incoming officials often seek to introduce new health agendas. However, such transitions were also seen as an opportunity to reposition and advance sodium reduction efforts in the light of a renewed agenda.

#### 3.5.3 Limited availability of low-sodium salt substitutes

Concerns about the availability of salt substitutes in Nigeria were frequently raised as a significant barrier to reach, acceptability, and accessibility of low-sodium salt substitutes. Also, inconsistent market presence and affordability concerns, especially in rural settings and local markets, were highlighted as potential barriers to widespread adoption and sustained use of salt substitutes.

#### 3.5.4 Proliferation of unhealthy food products

Participants described that the high and increasing reliance on importation of packaged and ultra-processed foods presents a major challenge to sodium control, given limited current capacity to verify or regulate the sodium content in these products.

## 4 Discussion

This qualitative study provides a robust examination of multi-stakeholder perspectives on the progress and challenges of implementation of sodium reduction policies in Nigeria, 3 years following the launch of the NMSAP. Compared with our previous formative interviews (21), this study highlights the need for stronger government agencies’ capacity and authority, national monitoring and accountability systems, infrastructure to support multisectoral coordination and inter-organizational networks, and financing—all of which likely underlie other factors that enable or hinder implementation progress (27). Taken together, these interim results indicate that while certain facilitators are present, they may not be sufficient to overcome persistent barriers which will require ongoing and new strategies. This work adds valuable insights to the limited but growing body of literature on policy implementation in low- and middle-income countries, which often operate within complex structural constraints and challenging socio-political environments (24).

Consistent with our earlier formative evaluation, we found generally low knowledge and awareness of excess sodium-related health risks, the recommended daily salt intake, and how to interpret sodium information on nutrition labels (21). Across stakeholder groups, there was a strong consensus on the need for sustained, multisectoral, and tailored awareness efforts to lower population sodium intake and improve health outcomes. Although a few existing campaigns and educational programs on salt reduction, primarily on social media and television, were mentioned by participants, these efforts were perceived as limited in reach and less prominent compared to campaigns targeting other dietary components, such as sugar. Stakeholders emphasized the importance of culturally appropriate strategies and called for consistent public education campaigns, driven by grassroots organizations, government bodies, and communication agencies, to educate the public on selecting low-salt foods, understanding recommended daily salt intake, and adopting healthy low-salt cooking practices. Suggested outreach channels included engaging trusted community figures such as traditional rulers and health influencers, as well as community platforms like town hall meetings and faith-based organizations, which have proven effective in promoting health initiatives within communities (28, 29). For example, South Africa’s Salt Watch campaign employed multiple forms of media in local languages (TV, radio and social media), featured a well-known doctor and media personality, distributed a reduced-salt recipe book, and engaged healthcare professionals to reinforce messages—resulting in reported shifts in salt-related behaviors (30). In school-based settings, participants in our study also recommended leveraging school assemblies, health clubs, and Parent-Teacher Association meetings to build on existing nutrition curricula and reinforce nutrition education among school-aged children (31).

A recurring theme throughout this study was the critical role of data infrastructure and regulatory frameworks in enabling effective sodium reduction policy implementation in Nigeria— an issue that mirrors broader challenges in health policy formulation and implementation across LMICs (32–34). Participants described the lack of baseline data on sodium intake and the sodium content of commonly consumed foods as key barriers to regulatory enforcement and the development of reformulation targets and national nutrient profiling systems. Encouragingly, recent national efforts have begun to fill these gaps, with new data from NaSS informing national sodium benchmarks and the development of Nigeria’s National Guidelines for Sodium Reduction (17). These guidelines outline evidence-based interventions and strategies to further strengthen policy implementation, including mandatory limits on sodium content in processed and packaged foods, as well as complementary measures such as front-of-package warning labels.

Consistent with findings from our previous research (21), current food labeling practices, including front-of-package labeling, were perceived as suboptimal, weakly enforced, and primarily present on imported food products. Trust in existing labels has been further eroded by concerns over their accuracy, legibility, and lack of contextual relevance. Key informants emphasized that for front-of-package labeling systems to be effective, mandatory labeling policies are essential, aligning with global evidence that such systems can positively influence both consumers purchasing behavior and industry reformulation practices (11, 35–37). In particular, the implementation of interpretive front-of-package labeling systems that highlight product unhealthiness, such as “high in” warning labels, Nutri-Score, and Health Star Rating, holds strong potential to reduce consumer demand for unhealthy packaged foods (11, 35, 37, 38).

Low-sodium, potassium-enriched salt substitutes have gained traction in a number of countries in recent years as an effective strategy to optimize sodium and potassium intake and reduce high blood pressure (39–42). Among recommended salt-reduction strategies, these substitutes require less effort from consumers and are likely to be sustainable for primary prevention, particularly in settings like Nigeria, where most dietary sodium is added during cooking or at the table (43). However, consistent with previous studies, we found that their acceptability and adoption are limited by safety concerns, individual taste preferences, and cost (44, 45). While several trials have demonstrated a comparable flavor (75% sodium chloride and 25% potassium chloride) to regular salt and minimal risk of potassium toxicity when used in cooking and food processing (39, 46, 47), further research is needed to identify strategies for implementation including to increase acceptability, sustainment, and scale-up of salt substitutes. As in other studies, participants perceived that reductions in sodium and increase in potassium can make the salt less salty in taste, potentially prompting consumers to use more (48). Affordability and availability also emerged as key barriers, especially in rural areas. Narrowing the price differences between regular and potassium-enriched salts through government subsidies and regulation could drive consumer demand and encourage manufacturers to improve availability (45). Stakeholders also recommended leveraging local herbs and traditional potassium-rich flavorings, which were viewed as culturally acceptable and readily accessible. However, more research is needed to assess the effectiveness of locally produced salt alternatives in reducing sodium intake and improving health outcomes.

Challenges in coordination, governance, and cross-sector communication were identified as overarching barriers to policy implementation, consistent with findings from other LMICs (27, 32, 49). Respondents highlighted the absence of a centralized mechanism to align priorities, track progress, and facilitate information-sharing across sectors, resulting in siloed efforts and missed opportunities for synergy. These challenges were compounded by unclear delegation of responsibilities across government agencies and the absence of sector-specific action plans with defined timelines for implementation. Inconsistent regulatory enforcement at multiple levels of government was another recurring concern, with respondents citing limited progress in advancing sodium reduction policies due to insufficient follow-up and accountability mechanisms. Without such systems in place, voluntary guidelines are unlikely to effectively drive product reformulation or improve population diets (11). Implementation of the policies was further constrained by limited leadership commitment, reflected in inadequate funding and resource allocation. Despite these challenges, participants pointed to promising developments, such as the creation of technical working groups, which may offer a foundation for stronger multisectoral coordination and more effective implementation moving forward.

Resistance from food manufacturers, stemming from perceived business risks and potential revenue losses, was a key concern that hindered implementation. This aligns with findings from our earlier formative work (21) and studies conducted in Malaysia, Australia, Thailand and other countries (32, 50–52). However, evaluations from Chile and Canada suggest that reformulation and initial production costs did not negatively affect profit margins nor significantly impact food prices across most food categories (53, 54). To address industry resistance and promote compliance with sodium reduction targets, current literature and Nigeria’s National Guidelines for Sodium Reduction recommend several promising strategies: (1) implementing mandatory, sector-wide regulations to ensure a level playing field; (2) collaborating with manufacturers and retailers on phased, incremental reformulation; and (3) providing training and technical assistance to manufacturers or retailers that may lack the in-house capacity to revise their recipes (17, 51, 55, 56).

Evidence from other countries indicates that mandatory, government-led sodium regulation is more effective than voluntary or industry-led initiatives (16, 57). For example, South Africa implemented phased mandatory regulation to limit salt levels across a wide range of processed foods, alongside public awareness campaigns, resulting in a 1.16 g/day reduction in population salt intake between 2015 and 2019, lowering the median intake to 6.1 g/day (58, 59). High compliance was largely attributed to “leveling the playing field,” with regulations applied evenly across all industry actors and supported by strong industry engagement (60). These findings offer important lessons for Nigeria, suggesting that scaling up mandatory regulations, alongside robust enforcement and public education, could achieve substantial and sustained reductions in population sodium intake.

Lessons emanating from this study point to four key areas of “good practice” that can inform ongoing and future sodium reduction efforts in Nigeria and other LMICs: (1) Co-production of evidence-informed policies and practices through strong collaboration between government and the scientific community; (2) Inclusive policy processes that foster meaningful multi-stakeholder engagement and local ownership; (3) Ongoing assessment and contextual analysis to better understand evolving environments and stakeholder dynamics; and (4) Alignment of activities with government priorities and guidelines to support effective rollout and long-term sustainability of sodium reduction policies (13, 15). These lessons are particularly relevant for LMICs in the early to middle stages of sodium reduction policy implementation and aiming to move from planning to action.

This study has several noteworthy strengths. First, we gathered perspectives from a wide range of stakeholders, including representatives from multiple organizations involved in the implementation of NMSAP sodium-related policies, as well as consumers. This enhances the relevance of our findings for policymakers, aiming to strengthen multisectoral nutrition policy in diverse settings. Second, our application of established implementation science frameworks helped structure our analysis and address the need for clearer conceptualization of the multilevel and multisectoral complexities that characterize policy implementation in LMICs, such as Nigeria. Third, the prospective repeated cross-sectional design, with data collection at multiple time points during pre-implementation (our formative evaluation) and implementation phases (including a three-year follow-up and a planned five-year follow-up), allowed for a rich, dynamic understanding of the implementation process, including progress made and key influencing factors.

However, the study also has limitations. Data collection was limited to three states representing three of the six geopolitical zones, and most respondents were from the Federal Capital Territory, which may limit the generalizability of findings. Representation from food manufacturers and retailers was limited, as securing interviews with these groups was challenging because contacts and interviews were difficult to obtain. As such, much of the information on industry perspectives was second-hand, based on reports from other informants who had interacted with the sector.

### 4.1 Future directions and implications for policy and practice

Since the completion of data collection for this study, the Federal Ministry of Health and Social Welfare has taken significant steps to strengthen multisectoral coordination for sodium reduction. In February 2024, the Ministry established a National Technical Working Group on Sodium Reduction comprising representatives from government, the scientific and health professional communities (including members of the Nigeria Sodium Study [NaSS]), civil society organizations, and international development partners. The group adopted a four-pronged approach, focusing on education, advocacy, benchmarking, and policy/guideline development, to align cross-sectoral efforts and guide the implementation and monitoring of sodium reduction initiatives.

Data from the NaSS has already played a pivotal role in advancing national sodium reduction efforts. It informed the establishment of sodium benchmarks for a range of processed and packaged foods, supported the development of Nigeria’s National Guidelines for Sodium Reduction (17) (See Supplementary Figure 1), and is guiding the forthcoming Front-of-Pack Labeling Policy. A key next step for the Technical Working Group, in collaboration with government agencies such as the National Agency for Food and Drug Administration and Control (NAFDAC), is to co-develop feasible implementation strategies to address existing barriers.

## 5 Conclusion

Understanding how and why government policies aimed at regulating unhealthy diets, particularly high sodium intake, have not been implemented as intended, and identifying where existing and new strategies need strengthening, is a critical step toward preventing and controlling diet-related CVD risk factors at the population level. Achieving NMSAP’s goal of reducing salt intake by 30% will depend on coordinated action to overcome implementation barriers and leverage key facilitators. These findings underscore the importance of embedding implementation science principles into national sodium reduction efforts to bridge the gap between policy and practice.

## Data Availability

The original contributions presented in the study are included in the article/Supplementary material, further inquiries can be directed to the corresponding author/s.
