# Supplementary figures and images for: Assessing the implementation of national sodium reduction policies in Nigeria: an interim qualitative evaluation of stakeholder perspectives
Source: Front Nutr. 2026 Mar 26;13:1704402. doi: 10.3389/fnut.2026.1704402 (PMC13061676; doi:10.3389/fnut.2026.1704402)

**Supplemental Figure 1. Guideline Development Process**

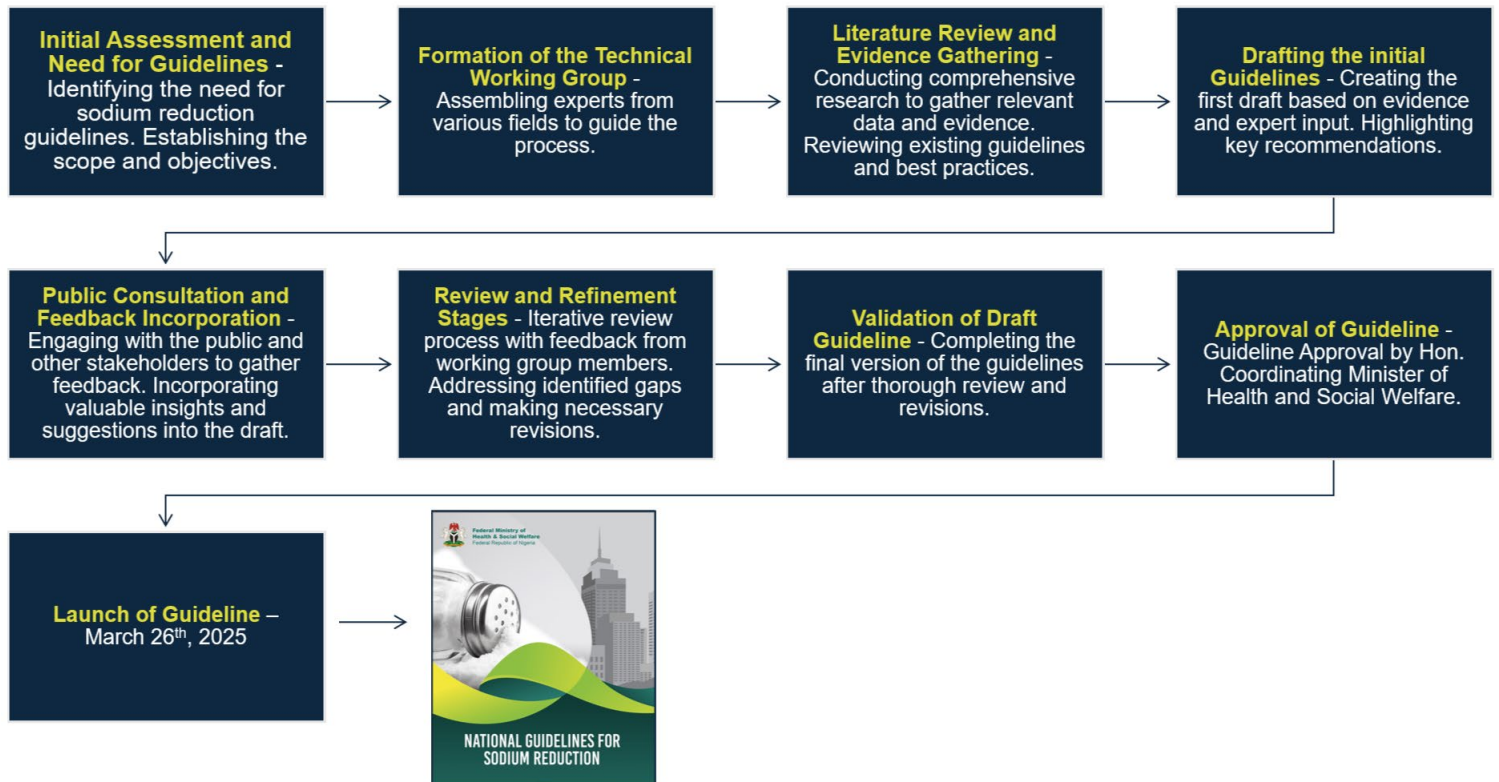

Supplement: Supplementary file 1 [file Image_1.pdf]
